# The Effects of Probiotic Supplementation on Opioid-Related Disorder in Patients under Methadone Maintenance Treatment Programs

**DOI:** 10.1155/2022/1206914

**Published:** 2022-03-30

**Authors:** Nader Molavi, Morad Rasouli-Azad, Hamed Mirzaei, Amir Hassan Matini, Hamid Reza Banafshe, Marjan Valiollahzadeh, Majid Hassanzadeh, Ahmad Reza Saghazade, Samira Abbaszadeh-Mashkani, Peyman Mamsharifi, Amir Ghaderi

**Affiliations:** ^1^Department of Addiction Studies, School of Medical, Kashan University of Medical Sciences, Kashan, Iran; ^2^International Center for Comparative Criminology, University of Montreal, Montreal, Canada; ^3^Research Center for Biochemistry and Nutrition in Metabolic Diseases, Kashan University of Medical Sciences, Kashan, Iran; ^4^Department of Clinical Pathology, Kashan University of Medical Sciences, Kashan, Iran; ^5^Physiology Research Center, Kashan University of Medical Sciences, Kashan, Iran; ^6^Student Research Committee, Kashan University of Medical Sciences, Kashan, Iran; ^7^Student Research Committee, Shahid Beheshti University of Medical Sciences, Tehran, Iran; ^8^Trauma Nursing Research Center, Faculty of Nursing and Midwifery, Kashan University of Medical Sciences, Kashan, Iran; ^9^Department of Psychology, Allameh Tabataba'i University, Tehran, Iran; ^10^Clinical Research Development Unit-Matini/Kargarnejad Hospital, Kashan University of Medical Sciences, Kashan, Iran

## Abstract

**Introduction:**

Patients under methadone maintenance treatment programs (MMTPs) are susceptible to numerous complications (e.g., mental and metabolic disorders). This study evaluated the effects of probiotics on clinical symptoms, biomarkers of oxidative stress, inflammation, insulin resistance, and serum lipid content in patients receiving MMTPs.

**Materials and Methods:**

A randomized, double-blind, placebo-controlled trial was conducted among 70 patients receiving MMTPs to receive either 1.8 × 10^9^ CFU/day probiotics (*n* = 35) or placebo (*n* = 35) for 12 weeks. Clinical symptoms and metabolic profiles were measured before and after the intervention in patients receiving MMTPs.

**Results:**

Compared with the placebo group, probiotic supplementation resulted in a significant improvement in the severity of depression (*P* < 0.05). In addition, probiotic administration significantly decreased fasting plasma glucose (FPG), total cholesterol, and low-density lipoprotein cholesterol (LDL cholesterol) (*P* < 0.05). Furthermore, probiotics resulted in a significant reduction in high-sensitivity C-reactive protein (hs-CRP) and a significant elevation in total antioxidant capacity (TAC) and total glutathione (GSH) levels (*P* < 0.05).

**Conclusion:**

Treatment with probiotics for 12 weeks to patients receiving MMTPs had beneficial effects on symptoms of depression, as well as several metabolic profiles. Clinical Trial Registration: this study was registered in the Iranian website (https://www.irct.ir) for clinical trials registration (https://fa.irct.ir/trial/46363/IRCT20170420033551N9). The registration date is March 22, 2020.

## 1. Introduction

According to the United Nations [1], more than 180 million people around the world are suffering from drug addiction. One of the most worrying post-addiction problems is malnutrition in all walks of life, whether in industrial or nonindustrial human societies [2]. In addition, overall body mass has been shown to increase predominantly due to an increase in the proportion of body fat upon methadone treatment [3]. A national pilot study was conducted in Iran in 2002 to assess the feasibility of reducing drug-related adverse effects, leading to the establishment of private and public centers for methadone treatment [4]. Several reasons support the efficacy of methadone as an effective treatment for opioid use disorder, including cost-effectiveness and strong potential for managing the physical and psychological condition of opioid-like addiction [5]. Approximately 5,000 outpatient methadone maintenance treatment programs (MMTPs) and buprenorphine exist in Iran to treat opioid dependency, covering nearly half a million subjects [6]. It should be noted that there remain multiple unresolved challenges and questions despite many practical MMTPs. Following the implementation of MMTPs, opioid abusers experienced several metabolic (such as inflammation and oxidative stress) and mental (such as sleep, depression, and anxiety) disorders, which affect their quality of life and increase the frequent drug addiction [7, 8]. Treatment with methadone, compared with buprenorphine, was associated with increased frequency of metabolic syndrome. Both drugs are associated with obesity, overweight, and insulin resistance. Extended methadone treatment may cause altered triglycerides, blood pressure, HDL, fasting glucose, and hemoglobin A1C profiles [9]. In addition, previous reports on gut microbiota have explored the effects of drug use on bacterial composition and diversity. Patients under MMTPs altered gut microbiota compared with former and current drug users, likely due to effects of both dietary changes and direct intake of the opiate methadone, leading to enrichment for bacteria (e.g., *Biﬁdobacterium, Fusicatenibacter*, and *Lactobacillus*) and enhanced orexin-A [10].

Gut microbiota represents a diverse number of microorganisms in the digestive tracts, which influence host physiology, including the immune system and the central nervous system [11]. In addition, the gut microbiota is known to be related to opioid use, psychiatric disorders, and type 2 diabetes [12]. The gut microbiota can regulate cerebral performance and behavior by modulating several neurometabolic and neurochemical processes [13, 14]. Special attention has recently been paid to probiotics due to wide clinical purposes and health-promoting effects to manage several clinical conditions, such as chronic and acute gastrointestinal and non-gastrointestinal problems [15]. Probiotics have numerous health-promoting effects, and the gut microbiota are vital for the regulation of metabolic and mood processes [16]. Patients with multiple sclerosis showed an improvement in general health, depression, and anxiety and stress scales following twelve weeks of probiotic supplementation in a study by Kouchaki et al. [17]. The results of a meta-analysis in randomized control trials indicated a reduction in depression and anxiety levels in patients supplemented with probiotics [18]. Nevertheless, there are reports on the lack of effect of probiotics on mental health outcomes in obese pregnant women [19]. A systematic review and meta-analysis showed that malondialdehyde level, C-reactive protein, interleukin-10, and Hamilton Depression Scale were positively improved in psychiatric patients supplemented with probiotics [20]. Further, psychiatric patients treated with probiotics exhibited improved levels of HDL cholesterol, VLDL, triglycerides, insulin resistance, insulin, malondialdehyde, and C-reactive protein. [21]. Beneficial activities have been reported for various probiotics, including enhanced absorption of nutrients, overexpression of brain-derived neurotrophic factor (BDNF), decreased levels of pro-inflammatory cytokines, free radical scavenging potential, overproduction of gamma-aminobutyric acid (GABA), regulation of critical neurotransmitters, and antioxidant activities. These activities may play a pivotal role in the pathology and physiology of metabolic and mental health processes [22–26].

A literature review revealed limited information on the effects of probiotic administration on depression, anxiety, sleep quality, lipid profiles, insulin sensitivity, inflammatory factors, and oxidative stress biomarkers in opioid-related disorder. Hence, the present research aimed to evaluate the influential activities of probiotics on clinical symptoms, biomarkers of oxidative stress, inflammation, insulin resistance, and serum lipid content among MMTP patients.

## 2. Materials and Methods

### 2.1. Participants

The study was conducted at the substance abuse Soltan Mirahmad Clinic, Kashan, Iran, between April 2020 and July 2020. This randomized, double-blind, placebo-controlled trial was registered in the Iranian Clinical Trials Registration of clinical trials (https://fa.irct.ir/trial/46363/IRCT20170420033551N9). The study protocol was approved by the Ethics Committee of Kashan University of Medical Sciences (approval code: IR.KAUMS.MEDNT.REC.1398.116) and was conducted according to the Declaration of Helsinki. Informed consent was obtained from all patients prior to the enrollment. All informed consent forms were reviewed by the Research Ethics Committee of KAUMS.

### 2.2. Inclusion/Exclusion Criteria

#### 2.2.1. Inclusion Criteria

1. Patients receiving MMTPs.
2. Aged 18 to 60 years.
3. Opioid use disorder as diagnosed by DSM-IV criteria.

#### 2.2.2. Exclusion Criteria

1. Unwillingness to cooperate.
2. Taking probiotic, multivitamin-mineral, and antioxidant supplements during the last 3 months before the intervention.
3. Positive urine tests indicating the use of any substance or methamphetamine during intervention.
4. Current severe depression and mania, and psychosis as determined by a study physician.
5. Metabolic diseases.
6. Cardiovascular disorder.

### 2.3. Study Design

In this randomized, double-blind, placebo-controlled trial, after balanced block randomization, patients were assigned to receive either probiotic (*n* = 35) or placebo (*n* = 35) for 12 weeks during receiving MMTPs. Probiotics contained four viable and freeze-dried strains: *Lactobacillus acidophilus, Bifidobacterium bifidum, Bifidobacterium longum, and Bifidobacterium lactis* (1.8 × 10^9^ CFU/g each; capsule). Probiotic supplements and placebos were produced by the LactoCare®, Takgene Pharma Company (Tehran, Iran). Random numbers were generated with computer software (Stat Trek) by trained staff at the Soltan Mirahmad Clinic. Randomization and allocation were concealed from the researchers and patients until the final analyses were completed. A person at the substance abuse treatment clinic, who was not involved in the trial and was unaware of random sequences, assigned the participants to the numbered bottles of supplements. At the clinic, patients received a weekly dose of methadone in the form of syrup, about half of the daily dose in the clinic and the remainder at home.

### 2.4. Assessment of Outcomes

Mental health is an important indicator of the health status of patients under MMTPs. Therefore, mental health (depression, anxiety, and sleep quality) was considered as the primary outcome, and lipid profiles, insulin metabolism, inflammatory factors, and oxidative stress biomarkers were considered as secondary outcomes.

### 2.5. Clinical Signs

In this study, Beck's Depression Inventory (BDI), Beck's Anxiety Inventory (BAI), and Pittsburgh Sleep Quality Index (PSQI) were used to assess the levels of depression, anxiety, and sleep quality, respectively [27–29]. BDI is a 21-question and BAI is a 20-question inventory in which each question was scored between 0 and 4; higher scores indicate higher levels of depression and anxiety. The Persian versions of both inventories were validated in the previous studies [30, 31]. PSQI is an applicable instrument used to measure the pattern and quality of sleep. It differentiates poor from good sleep quality by measuring seven components of sleep (e.g., subjective sleep quality, sleep duration, sleep latency, habitual sleep efficiency, use of sleeping medications, sleep disturbances, and daytime dysfunction) over the last month [29].

### 2.6. Biochemical Assessment

At baseline and after the 12-week intervention, fasting blood (10 mL) was collected from each patient at the substance abuse treatment center (Soltan Mirahmad Clinic). Serum insulin levels were measured with an ELISA kit (DiaMetra, Milano, Italy) with intra- and inter-assay CVs below 5%. The homeostasis model of assessment, QUICKI, and HOMA-IR were assessed using the established formulas [32]. Enzymatic kits (Pars Azmun, Tehran, Iran) with inter- and intra-assay CVs of less than 5% were used to measure fasting plasma glucose and lipid parameters. Serum hs-CRP levels were determined by the commercial ELISA kit (LDN, Nordhorn, Germany) with inter- and intra-assay CVs below 7%. In addition, NO levels were assessed using the Griess method [33]. In addition, TAC levels were assessed using the ferric-reducing antioxidant power method developed by Benzie and Strain [34]. In addition, GSH and MDA levels were evaluated using Beutler et al.'s method and thiobarbituric acid reactive substance spectrophotometric test, respectively [35, 36]. CVs for plasma MDA, GSH, and TAC were less than 5%.

### 2.7. Statistical Analysis

The normality of data was assessed by the Shapiro–Wilk test using the Statistical Package for Social Science Version 22 (SPSS Inc., Chicago, Illinois). To detect the differences in anthropometric parameters between treatment groups, we used the independent-samples *t*-test and chi-square test. Multiple linear regression models were used to assess treatment effects on study outcomes (clinical sign parameters, lipid profiles, insulin sensitivity, inflammatory factors, and oxidative stress biomarkers) after adjusting for baseline levels of variables. The effect sizes were presented as the mean differences with 95% confidence intervals. *P* values <0.05 were considered statistically significant.

## 3. Results

**Figure 1:**
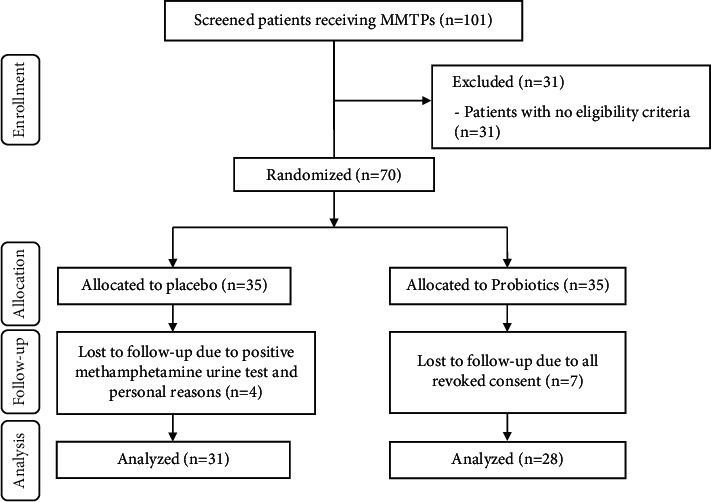
Summary of the patient flow diagram.

Of 101 screened patients with MMTPs, 70 patients were enrolled in the study and randomly assigned to either the intervention or control group to receive probiotics or placebo, respectively (35 people to each group). In the placebo group, four patients discontinued intervention due to positive methamphetamine urine test and personal reasons. In the intervention group, seven patients revoked their consents. Therefore, 59 patients (intervention (*n* = 28) and placebo (*n* = 31)) were analyzed. CONSORT flow diagram on the enrollment of patients in this study is shown in Figure 1. No side effects were ascertained following the administration of probiotics and placebos in patients receiving MMTPs. Patients' characteristics were analogous between the two groups (Table 1).

### 3.1. Clinical Signs

Compared with the placebo group, probiotics significantly decreased the level of depression (*β* 1.94; 95% CI, 0.14, 3.75; *P*=0.03), respectively. In addition, no significant differences were indicated between the two groups in terms of the Beck's Anxiety Inventory and Pittsburgh Sleep Quality Index (Table 2).

### 3.2. Metabolic Profiles

After the 3-month intervention, probiotic supplementation significantly decreased FPG (*β* 8.53 mg/dL; 95% CI, 3.32, 13.75; *P*=0.002), total cholesterol (*β* 15.14 mg/dL; 95% CI, 4.69, 25.60; *P*=0.005), and LDL cholesterol (*β* 14.51 mg/dL; 95% CI, 2.29, 26.74; *P*=0.02), compared with the placebo group. In addition, probiotic resulted in a significant reduction in hs-CRP (*β* 2.05 mg/L; 95% CI, 0.44, 3.66; *P*=0.01) and significant enhancement of TAC (*β* −110.02 mmol/L; 95% CI, −189.33, −30.71; *P*=0.007) and plasma GSH levels (*β* −66.33 *μ*mol/L; 95% CI, −107.13, −25.52; *P*=0.002) compared with the placebo. There was no significant effect of probiotic supplementation on insulin metabolism, HOMA-IR, QUICKI, triglycerides, VLDL and HDL cholesterol, malondialdehyde levels, and plasma nitric oxide (Table 3).

## 4. Discussion

Our study investigated the effects of probiotics on clinical symptoms, lipid profiles, insulin sensitivity, inflammatory factors, and oxidative stress biomarkers in patients receiving MMTPs. The results showed that a 12-week probiotic supplementation in patients receiving MMTPs had beneficial effects on depression, glycemic control, total and LDL cholesterol, hs-CRP serum level, total antioxidants, and plasma glutathione, no effect on anxiety, sleep quality, insulin levels, HOMA-IR, QUICKI, triglycerides, VLDL and HDL cholesterol, malondialdehyde levels, and plasma nitric oxide. Addiction can cause mental health disorder, metabolic disorder, and oral complications and may result in relapse to drug abuse and reduced addiction treatment success rate [7, 8, 37]. There seems to be an increasing volume of evidence of an association between metabolic disorder and periodontitis [38]. The periodontal disease represents a progressive destruction of tooth-supporting tissues. Recently, paraprobiotics are regarded as an adjunctive therapy to the nonsurgical scaling and root planning [39]. On the other hand, gut homeostasis plays an important role in human and animal health. The disruption of gut homeostasis enhances the risk of infections and represents potential side effect of opioid use. The effects of opioids in the gut homeostasis, both chronic and acute, include persistent constipation and might worsen pain, mental health signs, and metabolic syndrome [40–42]. In addition, the gut microbiota in rats treated with methamphetamine demonstrated that Bacillaceae and Ruminococcaceae were more abundant in the rats with methamphetamine-induced conditioned place preference [43]. So, gut microbial diversity might be shaped by addiction-associated behaviors and these can function as biological indicators to evaluate the health of people with a history of drug use. Also, disturbances in mental health parameters and metabolic profiles have previously been documented in opioid users and patients receiving MMTPs [7, 44]. This evidence suggested a potential link involving the gut microbiota, metabolism, and psychological factors that warrant consideration therapeutically. This is the first study to investigate the effects of probiotics on clinical symptoms, lipid profiles, insulin sensitivity, inflammatory factors, and biomarkers of oxidative stress in patients receiving MMTPs.

### 4.1. The Effect of Probiotics on Clinical Symptoms

The prevalence of mental disorders in patients receiving MMTPs is ten-fold higher than in the general population and two- to three-fold higher than those with substance abuse. In addition, the high prevalence of sleep disorders in patients receiving MMTPs has been estimated at 84% [45, 46]. A growing body of evidence has linked mental health symptoms to the gut microbiome, demonstrating that the latter might be modulating the gut-brain axis [47]. Disturbances in the microbiome have been implicated in both mental and physical illnesses. Signs of psychiatric disturbance, such as depression, anxiety, and autism, have been posited to be associated with disturbed gut microbiota [47, 48]. Opioid use significantly affects patients' diets and leads to malnutrition. Numerous studies have reported the effects of drug abuse on nutrition and nutritional deficiencies, and a beneficial effect of probiotics as a food or aromatic plant product in nutrition and diet has been advanced [49–51]. The gut microbiome as a therapeutic target for mental health disorder has been a focus in psychiatric research, among many others [52]. In our study, a 12-week probiotic supplementation in patients receiving MMTPs reduced depression scores but had no effect on anxiety scores and sleep quality. It has previously been reported that the use of probiotics has a beneficial effect on mental health and cognitive function [53]. Recent studies in animals and humans have shown that interventions involving intestinal microbiota, including probiotics, may be an effective treatment for mental health disorders [54, 55]. In an animal study, Li et al. reported that probiotics may reduce anxiety and depression [56]. Furthermore, young healthy women who received probiotics containing two strains of Lactobacillus helveticus and Bifidobacterium longum for 30 days experienced significant improvements in their mental health [57]. It has also been reported that long-term probiotic supplementation in rugby players may have beneficial effects on their sleep quality and muscle soreness during training and while playing games [58]. In a meta-analysis of 10 clinical trials, it was shown that probiotics had a marginal effect on mood in general [59]. In addition, no significant improvement was observed in patients with schizophrenia following treatment with *Lactobacillus rhamnosus* and *Bifidobacterium* supplements for 14 weeks [60]. These discrepancies might be due to differences in the strains used or the length of the studies. The exact mechanism of action of probiotics in the brain, and its effects on mental health disorders, has yet to be fully understood. It has been posited that the administration of probiotics may improve psychological symptoms by increasing plasma tryptophan levels, decreasing serotonin metabolite concentration in the frontal cortex, and decreasing dopamine metabolite concentrations in the amygdale [61].

### 4.2. The Effects of Probiotics on Lipid Profiles and Insulin Metabolism

Impaired insulin metabolism, hypertension, weight gain, and changes in serum lipids in patients receiving MMTPs may lead to long-term side effects, metabolic abnormalities, infections, chronic obstructive pulmonary disease, type 2 diabetes, and higher mortality [62–64]. Therefore, probiotics may be useful for lipid and blood glucose disorders due to their beneficial effects on lipid and glycemic parameters. Our study showed that consuming probiotics for 12 weeks in patients receiving MMTPs significantly reduced blood sugar, total cholesterol, and LDL cholesterol compared with placebo, but had no significant effect on insulin levels, HOMA-IR, QUICKI, triglycerides, and VLDL and HDL cholesterol. Although the information on the effects of probiotics on lipid and blood glucose profiles in patients receiving MMTPs is limited, several studies have evaluated these profiles in patients not receiving MMTPs. In 2019, Kijmanawat et al. studied GDM and controlled diet in 57 pregnant women. After four weeks of probiotic use, a significant improvement in fasting blood sugar and insulin and insulin resistance levels was observed [65]. In a meta-analysis in 2020, nine studies on the effects of probiotics on neurological diseases were analyzed, showing that probiotics had a significant positive effect on insulin, insulin resistance, triglycerides, and VLDL and HDL cholesterol [21]. In another study in patients with nonalcoholic fatty liver disease, liver enzymes and clinical signs were significantly reduced after 3 months of probiotic use, although no significant changes in triglyceride levels were observed. However, contrary to the results of our study, in this study no significant change in fasting blood sugar was reported [66]. The beneficial effects of probiotics on insulin sensitivity may be due to their effects on the expression of genes associated with fat accumulation and obesity. In addition, the production of short-chain fatty acids (SCFAs) following probiotic use, especially butyrate, stimulated the release of glucose-like peptide (GLP-1) from L intestinal cells and improved glucose tolerance.

### 4.3. The Effects of Probiotics on Inflammatory Biomarkers and Oxidative Stress

In patients receiving MMTPs, there is a loss of antioxidant capacity and an oxidative imbalance, reflected by increased reactive oxygen species (ROS) generation, matrix metalloproteinase activity, and inflammatory cytokines [67, 68]. Therefore, probiotics may have beneficial effects on inflammatory profiles and oxidative stress. This study showed that probiotic use for 12 weeks in patients receiving MMTPs significantly decreased serum hs-CRP levels and increased plasma levels of total antioxidants and glutathione compared with the placebo group, but had no effect on plasma nitric oxide and MDA levels. Probiotics had inhibitory effects on several oxidative stress factors in nervous system disorders in both human [17] and animal models [69–71]. Probiotics improved oxidative stress indices, such as SOD and MDA, in an animal model of Alzheimer's disease [70]. In another study in Alzheimer's disease patients, administration for 12 weeks of probiotics containing *Lactobacillus acidophilus, Lactobacillus casei, Bifidobacterium bifidum*, and *Lactobacillus fermentum* reduced MDA, but had no effect on TAC and GSH levels [72]. In a meta-analysis of 12 studies in 2020, Amirani et al. examined the effects of probiotics on metabolic factors in patients with psychiatric disorders, showing beneficial effects on CRP, IL-10, and MDA levels [20]. In addition, 12 weeks of probiotics and vitamin D administration in patients with schizophrenia increased total antioxidants and decreased MDA and hs-CRP [73]. Probiotics decrease oxidative stress via several mechanisms. These include direct antioxidant capacity, production of antioxidant metabolites, stimulation of host antioxidant activity, increased activity of antioxidant enzyme levels by acting on NF-*κ*B and Nrf2 gene expression, and decreased activity of ROS-producing enzymes such as NADPH oxidase [74–76]. In addition, probiotics may affect the expression of cytokines and inflammatory chemokines by acting on TLR receptors and cascading pathways and by acting on genes essential to the inflammatory mechanisms, such as NF*-κ*B [77].

### 4.4. Study Limitations

Long-term interventions may have better effects on other metabolic profiles, anxiety, and sleep quality. Also, we did not evaluate the dietary intakes of study participants; however, we requested the participants not to change their regular dietary intakes and physical activity. In addition, we did not evaluate craving, pain, urinary profiles, and relapses in patients under MMTPs.

## 5. Conclusion

Overall, our novel findings suggest that patients receiving MMTPs and undergoing 12-week probiotic administration experienced improvements in symptoms of depression, but no change in anxiety and sleep quality. In addition, our study showed that probiotics had beneficial effects on blood sugar, total cholesterol, LDL cholesterol, serum hs-CRP levels, total antioxidants, and plasma glutathione, no effect on insulin, HOMA-IR, QUICKI, triglyceride, HDL and VLDL cholesterol, and plasma dialdehyde and nitric oxide levels. Further evidence is needed to demonstrate the efficacy of probiotics in patients receiving MMTPs, better characterizing their mode of action.

## Figures and Tables

**Table 1:** Characteristics of patients receiving MMTPs^1^.

	Placebo (*n* = 31)	Probiotics (*n* = 28)	*P* ^2^
Age (year)	47.6 ± 8.4	44.4 ± 10.3	0.20
Age first experience of drug use (%)	20.6 ± 7.5	21.0 ± 7.6	0.83
*The first type of consumable (%)*			
Opium and opium ashes	15 (48.4)	16 (57.1)	0.34^†^
Heroin or cracked heroin	8 (25.8)	3 (10.7)	
Marijuana	6 (19.4)	6 (21.4)	
Methamphetamine	2 (6.5)	1 (3.6)	
Other different substances	0 (0)	2 (7.1)	
*Psychotropic medications (%)*			
Sedative hypnotics	2 (6.5)	4 (14.3)	0.59^†^
Antidepressants	3 (9.7)	2 (7.1)	
None	26 (83.9)	22 (78.6)	
Height (cm)	171.6 ± 15.4	173.9 ± 8.6	0.49
Weight at study baseline (kg)	72.5 ± 13.9	83.0 ± 23.5	0.04
Weight at the end of trial (kg)	72.6 ± 13.9	82.2 ± 22.4	0.05
BMI at study baseline (kg/m^2^)	25.1 ± 6.6	27.3 ± 6.6	0.21
BMI at the end of trial (kg/m^2^)	25.3 ± 7.7	27.0 ± 6.3	0.35
*Education (%)*			
Illiterate	2 (6.5)	3 (10.7)	0.34^†^
Elementary	13 (41.9)	13 (46.4)	
Intermediate	15 (48.4)	8 (28.6)	
Diploma	0 (0)	2 (7.1)	
High educated	1 (3.2)	2 (7.1)	
*Marital status (%)*			
Single	8 (25.8)	10 (35.7)	0.37^†^
Married	19 (61.3)	17 (60.7)	
Widow/divorced	4 (12.9)	1 (3.6)	
*Job (%)*			
Unemployed	10 (32.3)	8 (28.6)	0.91^†^
Employed	5 (6.1)	4 (14.3)	
Others	16 (51.6)	16 (57.1)	
MMT dose (mL/d)	19.5 ± 13.3	19.5 ± 6.6	0.98
In-person daily dosing (mL)	9.8 ± 6.7	9.9 ± 3.2	0.94
Take-home dosing (mL/weekly)	127.2 ± 86.7	126.8 ± 43.1	0.98
Duration of MMT (y)	7.5 ± 4.3	9.8 ± 5.3	0.08

^1^Data are mean ± SDs and percentage. ^2^Obtained from independent *t*-test. ^†^Obtained from the Pearson chi-square test.

**Table 2:** Means (±standard deviation) of clinical sign parameters at baseline and after the 12-week intervention in patients receiving MMTPs^1^.

Variables	Placebo (*n* = 31)		Probiotics (*n* = 28)		Difference in outcome measures between probiotics and placebo treatment groups^1^	
	Baseline	Week 12	Baseline	Week 12	*β* (95% CI)	*P* ^2^
BDI	20.5 ± 10.4	19.5 ± 10.3	25.2 ± 14.3	22.1 ± 13.2	1.94 (0.14, 3.75)	0.03
BAI	13.7 ± 10.3	12.2 ± 8.9	14.8 ± 10.0	11.2 ± 8.2	1.69 (−0.08, 3.48)	0.06
PSQI	4.0 ± 1.7	4.0 ± 1.6	5.3 ± 2.2	4.8 ± 1.9	0.007 (−0.58, 0.60)	0.98

Data are mean ± SDs. ^1^“Outcome measures” refers to the change in values of measures of interest between baseline and week 12. *β* (difference in the mean outcome measures between treatment groups (probiotic group = 1 and placebo group = 0)). ^2^Obtained from multiple regression models. BDI, Beck Depression Inventory; BAI, Beck Anxiety Inventory; PSQI, Pittsburgh Sleep Quality Index.

**Table 3:** Means (±standard deviation) of lipid profiles, insulin sensitivity, inflammatory factors, and oxidative stress biomarkers at baseline and after the 12-week intervention in patients receiving MMTPs^1^.

Variables	Placebo (*n* = 31)		Probiotics (*n* = 28)		Difference in outcome measures between probiotics and placebo treatment groups^1^	
	Baseline	Week 12	Baseline	Week 12	*β* (95% CI)	*P* ^2^
FPG (mg/dL)	90.3 ± 18.9	89.1 ± 21.0	86.4 ± 15.1	77.3 ± 11.5	8.53 (3.32, 13.75)	0.002
Insulin (*µ*IU/mL)	13.0 ± 4.4	11.7 ± 5.5	14.3 ± 5.0	10.3 ± 5.4	1.94 (−0.51, 4.40)	0.11
HOMA-IR	3.0 ± 1.1	2.6 ± 1.2	3.1 ± 1.5	2.1 ± 1.1	0.43 (−0.08, 0.96)	0.09
QUICKI	0.33 ± 0.02	0.34 ± 0.03	0.32 ± 0.02	0.34 ± 0.07	0.001 (−0.02, 0.02)	0.95
Triglycerides (mg/dL)	142.5 ± 84.4	141.7 ± 86.0	151.4 ± 65.3	142.0 ± 64.6	7.51 (−3.30, 18.33)	0.16
VLDL cholesterol (mg/dL)	28.5 ± 16.8	28.3 ± 17.2	30.2 ± 13.0	28.4 ± 12.9	1.50 (−0.66, 3.66)	0.16
Total cholesterol (mg/dL)	174.1 ± 47.1	172.4 ± 46.6	187.6 ± 57.5	168.5 ± 49.5	15.14 (4.69, 25.60)	0.005
LDL cholesterol (mg/dL)	101.6 ± 54.8	99.4 ± 52.4	116.0 ± 56.2	97.5 ± 48.7	14.51 (2.29, 26.74)	0.02
HDL cholesterol (mg/dL)	43.9 ± 10.4	44.6 ± 9.1	41.3 ± 10.1	42.5 ± 8.9	−0.51 (−3.82, 2.78)	0.75
Hs-CRP (mg/L)	6.2 ± 3.0	7.3 ± 3.9	6.0 ± 4.1	5.0 ± 4.1	2.05 (0.44, 3.66)	0.01
Total nitrite (*µ*mol/L)	50.0 ± 13.3	52.3 ± 13.6	52.4 ± 13.9	54.5 ± 14.7	0.48 (−2.47, 3.44)	0.74
TAC (mmol/L)	928.3 ± 174.5	941.5 ± 185.4	886.1 ± 184.2	1016.9 ± 143.1	−110.02 (−189.33, −30.71)	0.007
GSH (*µ*mol/L)	655.8 ± 168.4	691.6 ± 161.3	626.9 ± 149.8	728.4 ± 148.0	−66.33 (−107.13, −25.52)	0.002
MDA (*µ*mol/L)	2.8 ± 0.5	3.2 ± 0.4	2.7 ± 0.4	3.3 ± 0.4	−0.14 (−0.37, 0.08)	0.20

Data are mean ± SD. ^1^“Outcome measures” refers to the change in values of measures of interest between baseline and week 12. *β* (difference in the mean outcome measures between treatment groups (probiotic group = 1 and placebo group = 0)). ^2^Obtained from multiple regression models. FPG, fasting plasma glucose; GSH, total glutathione; HOMA-IR, homeostasis model of assessment insulin resistance; HDL cholesterol, high-density lipoprotein cholesterol; Hs-CRP, high-sensitivity C-reactive protein; LDL cholesterol, low-density lipoprotein cholesterol; NO, nitric oxide; QUICKI, quantitative insulin sensitivity check index; VLDL cholesterol, very low-density lipoprotein cholesterol; TAC, total antioxidant capacity; MDA, malondialdehyde.

## Data Availability

The datasets generated and/or analyzed during this study are not publicly available because the intellectual property is owned by the funding body. They may be available from the corresponding author on reasonable request containing the approval from the associated funding body.

## Abbreviations

FPG: Fasting plasma glucose
GSH: Total glutathione
HOMA-IR: Homeostasis model of assessment insulin resistance
HDL cholesterol: High-density lipoprotein cholesterol
Hs-CRP: High-sensitivity C-reactive protein
LDL cholesterol: Low-density lipoprotein cholesterol
NO: Nitric oxide
QUICKI: Quantitative insulin sensitivity check index
VLDL cholesterol: Very low-density lipoprotein cholesterol
TAC: Total antioxidant capacity
MDA: Malondialdehyde
BDI: Beck Depression Inventory
BAI: Beck Anxiety Inventory
PSQI: Pittsburgh Sleep Quality Index.

## References

[1] Drugs UNOo Crime (2019). World drug report 2019: 35 million people worldwide suffer from drug use disorders while only 1 in 7 people receive treatment. https://www.unodc.org/unodc/en/frontpage/2019/June/world-drug-report-2019_-35-million-people-worldwide-suffer-from-drug-use-disorders-while-only-1-in-7-people-receive-treatment.html.

[2] Mousali A. A., Bashirian S., Barati M. (2021). Factors affecting substance use relapse among Iranian addicts. *Journal of Education and Health Promotion*.

[3] Sadek G. E., Chiu S., Cernovsky Z. Z. (2016). Body composition changes associated with methadone treatment. *International Journal of High Risk Behaviors & Addiction*.

[4] Malekinejad M., Vazirian M. (2012). Transition to injection amongst opioid users in Iran: implications for harm reduction. *International Journal of Drug Policy*.

[5] Khodabandeh S., Loripoor M., Hossinrezaei H. (2008). The comparison religious and familial characteristics between abuser drug quiting addiction in Mahyar rehabilitation center of Isfahan and control group. *Journal of Rafsanjan University of Medical Sciences*.

[6] Lankarani K. B., Afshari R. (2014). Alcohol consumption in Iran. *Lancet*.

[7] Molavi N., Ghaderi A., Banafshe H. R. (2020). Examining metabolic profiles in opioid-dependent patients. *International Journal of Medical Toxicology and Forensic Medicine*.

[8] Le T. A., Le M. Q. T., Dang A. D. (2019). Multi-level predictors of psychological problems among methadone maintenance treatment patients in difference types of settings in Vietnam. *Substance Abuse Treatment, Prevention, and Policy*.

[9] Elman I., Howard M., Borodovsky J. T. (2020). Metabolic and addiction indices in patients on opioid agonist medication-assisted treatment: a comparison of buprenorphine and methadone. *Scientific Reports*.

[10] Li Q., Chen S., Liu K. (2020). Differences in gut microbial diversity are driven by drug use and drug cessation by either compulsory detention or methadone maintenance treatment. *Microorganisms*.

[11] Fung T. C., Olson C. A., Hsiao E. Y. (2017). Interactions between the microbiota, immune and nervous systems in health and disease. *Nature Neuroscience*.

[12] Barengolts E., Green S. J., Eisenberg Y. (2018). Gut microbiota varies by opioid use, circulating leptin and oxytocin in African American men with diabetes and high burden of chronic disease. *PLoS One*.

[13] Bravo J. A., Julio-Pieper M., Forsythe P. (2012). Communication between gastrointestinal bacteria and the nervous system. *Current Opinion in Pharmacology*.

[14] Douglas-Escobar M., Elliott E., Neu J. (2013). Effect of intestinal microbial ecology on the developing brain. *JAMA Pediatrics*.

[15] Zuccotti G. V., Meneghin F., Raimondi C. (2008). Probiotics in clinical practice: an overview. *Journal of International Medical Research*.

[16] Desbonnet L., Garrett L., Clarke G., Kiely B., Cryan J. F., Dinan T. G. (2010). Effects of the probiotic bifidobacterium infantis in the maternal separation model of depression. *Neuroscience*.

[17] Kouchaki E., Tamtaji O. R., Salami M. (2017). Clinical and metabolic response to probiotic supplementation in patients with multiple sclerosis: a randomized, double-blind, placebo-controlled trial. *Clinical Nutrition*.

[18] Chao L., Liu C., Sutthawongwadee S. (2020). Effects of probiotics on depressive or anxiety variables in healthy participants under stress conditions or with a depressive or anxiety diagnosis: a meta-analysis of randomized controlled trials. *Frontiers in Neurology*.

[19] Dawe J. P., McCowan L. M. E., Wilson J., Okesene-Gafa K. A. M., Serlachius A. S. (2020). Probiotics and maternal mental health: a randomised controlled trial among pregnant women with obesity. *Scientific Reports*.

[20] Amirani E., Milajerdi A., Mirzaei H. (2020). The effects of probiotic supplementation on mental health, biomarkers of inflammation and oxidative stress in patients with psychiatric disorders: a systematic review and meta-analysis of randomized controlled trials. *Complementary Therapies in Medicine*.

[21] Tamtaji O. R., Milajerdi A., Reiner Ž. (2020). A systematic review and meta-analysis: the effects of probiotic supplementation on metabolic profile in patients with neurological disorders. *Complementary Therapies in Medicine*.

[22] Ait-Belgnaoui A., Colom A., Braniste V. (2014). Probiotic gut effect prevents the chronic psychological stress-induced brain activity abnormality in mice. *Neuro-Gastroenterology and Motility*.

[23] Luo J., Wang T., Liang S., Hu X., Li W., Jin F. (2014). Ingestion of lactobacillus strain reduces anxiety and improves cognitive function in the hyperammonemia rat. *Science China Life Sciences*.

[24] Li S., Zhao Y., Zhang L. (2012). Antioxidant activity of Lactobacillus plantarum strains isolated from traditional Chinese fermented foods. *Food Chemistry*.

[25] Dhakal R., Bajpai V. K., Baek K.-H. (2012). Production of gaba (*γ*-aminobutyric acid) by microorganisms: a review. *Brazilian Journal of Microbiology*.

[26] Jumpertz R., Le D. S., Turnbaugh P. J. (2011). Energy-balance studies reveal associations between gut microbes, caloric load, and nutrient absorption in humans. *American Journal of Clinical Nutrition*.

[27] Beck A. T., Steer R. A., Carbin M. G. (1988). Psychometric properties of the beck depression inventory: twenty-five years of evaluation. *Clinical Psychology Review*.

[28] Beck A. T., Ward C., Mendelsohn M., Mock J., Erbaugh J. (1961). An inventory for measuring depression. *Archives of General Psychiatry*.

[29] Buysse D. J., Reynolds C. F., Monk T. H., Berman S. R., Kupfer D. J. (1989). The pittsburgh sleep quality index: a new instrument for psychiatric practice and research. *Psychiatry Research*.

[30] Rafiei M., Seifi A. (2013). An investigation into the reliability and validity of beck anxiety inventory among the university students. *Journal of Thought & Behavior in Clinical Psychology*.

[31] Vasegh S., Baradaran N. (2014). Using the Persian-language version of the beck depression inventory-II (BDI-II-Persian) for the screening of depression in students. *Journal of Nervous and Mental Disease*.

[32] Pisprasert V., Ingram K. H., Lopez-Davila M. F., Munoz A. J., Garvey W. T. (2013). Limitations in the use of indices using glucose and insulin levels to predict insulin sensitivity. *Diabetes Care*.

[33] Tatsch E., Bochi G. V., Pereira R. D. S. (2011). A simple and inexpensive automated technique for measurement of serum nitrite/nitrate. *Clinical Biochemistry*.

[34] Benzie I. F. F., Strain J. J. (1996). The ferric reducing ability of plasma (FRAP) as a measure of “antioxidant power”: the FRAP assay. *Analytical Biochemistry*.

[35] Beutler E., Gelbart T. (1985). Plasma glutathione in health and in patients with malignant disease. *Journal of Laboratory and Clinical Medicine*.

[36] Janero D. R. (1990). Malondialdehyde and thiobarbituric acid-reactivity as diagnostic indices of lipid peroxidation and peroxidative tissue injury. *Free Radical Biology and Medicine*.

[37] Shekarchizadeh H., Khami M. R., Mohebbi S. Z., Ekhtiari H., Virtanen J. I. (2019). Oral health status and its determinants among opiate dependents: a cross-sectional study. *BMC Oral Health*.

[38] Chandra R. V., Srinivas G., Reddy A. A. (2013). Locally delivered antioxidant gel as an adjunct to nonsurgical therapy improves measures of oxidative stress and periodontal disease. *Journal of Periodontal & Implant Science*.

[39] Butera A., Gallo S., Pascadopoli M. (2022). Paraprobiotics in non-surgical periodontal therapy: clinical and microbiological aspects in a 6-month follow-up domiciliary protocol for oral hygiene. *Microorganisms*.

[40] Wang F., Roy S. (2017). Gut homeostasis, microbial dysbiosis, and opioids. *Toxicologic Pathology*.

[41] Akbarali H. I., Dewey W. L. (2017). The gut-brain interaction in opioid tolerance. *Current Opinion in Pharmacology*.

[42] Clapp M., Aurora N., Herrera L., Bhatia M., Wilen E., Wakefield S. (2017). Gut microbiota’s effect on mental health: the gut-brain axis. *Clinical Practice*.

[43] Ning T., Gong X., Xie L., Ma B. (2017). Gut microbiota analysis in rats with methamphetamine-induced conditioned place preference. *Frontiers in Microbiology*.

[44] Abousaeedi H., Hosseini O. R., Bidaki R. (2016). Viral and psychiatric disorders in methadone maintenance therapy (MMT) clients. *Thrita*.

[45] Callaly T., Trauer T., Munro L., Whelan G. (2001). Prevalence of psychiatric disorder in a methadone maintenance population. *Australian and New Zealand Journal of Psychiatry*.

[46] Stein M. D., Herman D. S., Bishop S. (2004). Sleep disturbances among methadone maintained patients. *Journal of Substance Abuse Treatment*.

[47] Nasrallah H. A. (2018). It takes guts to be mentally ill: microbiota and psychopathology. *Current Psychiatry*.

[48] Pastis I., Saeed S. A., Muthukanagaraj P. (2019). Gut microbiota and its implications for psychiatry: a review of 3 studies. *Current Psychiatry*.

[49] Gupta S., Abu-Ghannam N. (2012). Probiotic fermentation of plant based products: possibilities and opportunities. *Critical Reviews in Food Science and Nutrition*.

[50] Brown A. C., Valiere A. (2004). Probiotics and medical nutrition therapy. *Nutrition in Clinical Care: An Official Publication of Tufts University*.

[51] Song D., Ibrahim S., Hayek S. (2012). Recent application of probiotics in food and agricultural science. *Probiotics*.

[52] Butler M. I., Mörkl S., Sandhu K. V., Cryan J. F., Dinan T. G. (2019). The gut microbiome and mental health: what should we tell our patients?: le microbiote intestinal et la santé mentale: que devrions-nous dire à nos patients?. *Canadian Journal of Psychiatry*.

[53] Ansari F., Pourjafar H., Tabrizi A., Homayouni A. (2020). The effects of probiotics and prebiotics on mental disorders: a review on depression, anxiety, Alzheimer, and autism spectrum disorders. *Current Pharmaceutical Biotechnology*.

[54] Wang H., Lee I.-S., Braun C., Enck P. (2016). Effect of probiotics on central nervous system functions in animals and humans: a systematic review. *Journal of Neurogastroenterology and Motility*.

[55] Kazemi A., Noorbala A. A., Azam K., Eskandari M. H., Djafarian K. (2019). Effect of probiotic and prebiotic vs placebo on psychological outcomes in patients with major depressive disorder: a randomized clinical trial. *Clinical Nutrition*.

[56] Li N., Wang Q., Wang Y. (2018). Oral probiotics ameliorate the behavioral deficits induced by chronic mild stress in mice via the gut microbiota-inflammation axis. *Frontiers in Behavioral Neuroscience*.

[57] Chamari M., Djazayery A., Jalali M. (2008). The effect of daily consumption of probiotic and conventional yoghurt on some oxidative stress factors in plasma of young healthy women. *ARYA Atherosclerosis Journal*.

[58] Harnett J. E., Pyne D. B., McKune A. J., Penm J., Pumpa K. L. (2021). Probiotic supplementation elicits favourable changes in muscle soreness and sleep quality in rugby players. *Journal of Science and Medicine in Sport*.

[59] Ng Q. X., Peters C., Ho C. Y. X., Lim D. Y., Yeo W.-S. (2018). A meta-analysis of the use of probiotics to alleviate depressive symptoms. *Journal of Affective Disorders*.

[60] Dickerson F. B., Stallings C., Origoni A. (2014). Effect of probiotic supplementation on schizophrenia symptoms and association with gastrointestinal functioning: a randomized, placebo-controlled trial. *The Primary Care Companion for CNS Disorders*.

[61] Desbonnet L., Garrett L., Clarke G., Bienenstock J., Dinan T. G. (2008). The probiotic bifidobacteria infantis: an assessment of potential antidepressant properties in the rat. *Journal of Psychiatric Research*.

[62] Cousins G., Teljeur C., Motterlini N., McCowan C., Dimitrov B. D., Fahey T. (2011). Risk of drug-related mortality during periods of transition in methadone maintenance treatment: a cohort study. *Journal of Substance Abuse Treatment*.

[63] Maruyama A., Macdonald S., Borycki E., Zhao J. (2013). Hypertension, chronic obstructive pulmonary disease, diabetes and depression among older methadone maintenance patients in British Columbia. *Drug and Alcohol Review*.

[64] Vallecillo G., Robles M. J., Torrens M. (2018). Metabolic syndrome among individuals with heroin use disorders on methadone therapy: prevalence, characteristics, and related factors. *Substance Abuse*.

[65] Kijmanawat A., Panburana P., Reutrakul S., Tangshewinsirikul C. (2019). Effects of probiotic supplements on insulin resistance in gestational diabetes mellitus: a double-blind randomized controlled trial. *Journal of Diabetes Investigation*.

[66] Manzhalii E., Virchenko O., Falalyeyeva T., Beregova T., Stremmel W. (2017). Treatment efficacy of a probiotic preparation for non-alcoholic steatohepatitis: a pilot trial. *Journal of Digestive Diseases*.

[67] Rajagopalan S., Meng X. P., Ramasamy S., Harrison D. G., Galis Z. S. (1996). Reactive oxygen species produced by macrophage-derived foam cells regulate the activity of vascular matrix metalloproteinases in vitro: implications for atherosclerotic plaque stability. *Journal of Clinical Investigation*.

[68] Salarian A., Kadkhodaee M., Zahmatkesh M. (2018). Opioid use disorder induces oxidative stress and inflammation: the attenuating effect of methadone maintenance treatment. *Iranian Journal of Psychiatry*.

[69] Davari S., Talaei S. A., Alaei H., salami M. (2013). Probiotics treatment improves diabetes-induced impairment of synaptic activity and cognitive function: behavioral and electrophysiological proofs for microbiome-gut-brain axis. *Neuroscience*.

[70] Athari Nik Azm S., Djazayeri A., Safa M. (2018). Lactobacilli and bifidobacteria ameliorate memory and learning deficits and oxidative stress in *β*-amyloid (1–42) injected rats. *Applied Physiology Nutrition and Metabolism*.

[71] Xiao J., Li S., Sui Y. (2014). Lactobacillus casei-01 facilitates the ameliorative effects of proanthocyanidins extracted from lotus seedpod on learning and memory impairment in scopolamine-induced amnesia mice. *PLoS One*.

[72] Akbari E., Asemi Z., Daneshvar Kakhaki R. (2016). Effect of probiotic supplementation on cognitive function and metabolic status in Alzheimer’s disease: a randomized, double-blind and controlled trial. *Frontiers in Aging Neuroscience*.

[73] Ghaderi A., Banafshe H. R., Mirhosseini N. (2019). Clinical and metabolic response to vitamin D plus probiotic in schizophrenia patients. *BMC Psychiatry*.

[74] Wang Y., Wu Y., Wang Y. (2017). Antioxidant properties of probiotic bacteria. *Nutrients*.

[75] Mishra V., Shah C., Mokashe N., Chavan R., Yadav H., Prajapati J. (2015). Probiotics as potential antioxidants: a systematic review. *Journal of Agricultural and Food Chemistry*.

[76] Gao D., Gao Z., Zhu G. (2013). Antioxidant effects of lactobacillus plantarum via activation of transcription factor Nrf2. *Food & Function*.

[77] Mariman R., Kremer B., van Erk M., Lagerweij T., Koning F., Nagelkerken L. (2012). Gene expression profiling identifies mechanisms of protection to recurrent trinitrobenzene sulfonic acid colitis mediated by probiotics. *Inflammatory Bowel Diseases*.

